# Drivers of U.S. toxicological footprints trajectory 1998–2013

**DOI:** 10.1038/srep39514

**Published:** 2016-12-21

**Authors:** S. C. L. Koh, T. Ibn-Mohammed, A. Acquaye, K. Feng, I. M. Reaney, K. Hubacek, H. Fujii, K. Khatab

**Affiliations:** 1 Centre for Energy, Environment and Sustainability, The University of Sheffield, Sheffield, S10 1FL, UK; 2 Advanced Resource Efficiency Centre, The University of Sheffield, Sheffield, S10 1FL, UK; 3 Kent Business School, University of Kent, Canterbury, CT2 7PE, UK; 4 Department of Geographical Sciences, University of Maryland, College Park, Maryland 20742, USA; 5 Departments of Materials Science and Engineering, The University of Sheffield, Sheffield, S1 3JD, UK; 6 Department of Environmental Studies, Masaryk University, Brno, Czech Republic; 7 Graduate School of Fisheries and Environmental Sciences, Nagasaki University 1–14 Bunkyo-machi, Japan; 8 Centre for Health and Social Care Research, Sheffield Hallam University, Sheffield, S10 2BP, UK

## Abstract

By exploiting data from the Toxic Release Inventory of the United States, we have established that the toxicological footprint (TF) increased by 3.3% (88.4 Mt) between 1998 and 1999 and decreased by 39% (1088.5 Mt) between 1999 and 2013. From 1999 to 2006, the decreasing TF was driven by improvements in emissions intensity (i.e. gains in production efficiency) through toxic chemical management options: cleaner production; end of pipe treatment; transfer for further waste management; and production scale. In particular, the mining sector reduced its TF through outsourcing processes. Between 2006 and 2009, decreasing TF was due to decrease in consumption volume triggered by economic recession. Since 2009, the economic recovery increased TF, overwhelming the influence of improved emissions intensity through population growth, consumption and production structures. Accordingly, attaining a less-toxic economy and environment will be influenced by a combination of gains in production efficiency through improvement in emissions mitigation technologies and changes in consumption patterns. Overall, the current analysis highlights the structural dynamics of toxic chemical release and would inform future formulation of effective mitigation standards and management protocols towards the detoxification of the environment.

Globally, supporting evidence and wider international recognition of the effect of climate change^1^ has demonstrated a need to mitigate atmospheric CO_2_ concentration^2^,^3^ and prompted a range of international, regional and national energy and emissions policies targeting energy intensive sectors^4^,^5^. However, long-lasting and pervasive toxic chemical release impact on climate change and alter global and local development. Toxic emissions impact on soils and water bodies, affect ecosystems and are detrimental to human health, quality of life and wellbeing^6^,^7^. A new study published in the latest issue of environmental toxicology and chemistry^8^ highlighted among other factors that human actions (including mitigation of and adaptation to impacts of global climate change, GCC) may have as much influence on the fate and distribution of chemical pollutants as GCC. As such, climate change has the potential to alter human chemical exposures by changing how chemicals move and transform in the environment. The Intergovernmental Forum on Chemical Society (IFCS)^9^ reported that the physical changes in temperature, wind and rainfall caused by climate change affect the distribution and breakdown of chemicals in complex ways. This effect on human exposure will vary according to the properties of specific chemicals and chemical combinations, soil and water conditions, wind patterns, topography, land use, level of development and human demographics.

In the light of the above, gaining an understanding of the trends in toxic chemical release based on a robust analytical framework is therefore pertinent and this study draws on the Toxics Release Inventory (TRI) of the United States (U.S.), to explore these issues. Numerous studies have employed the TRI datasets for environmental analyses. Yet, despite the potentially significant implications for the wider U.S. environmental policy, there has been no quantitative analysis that provides a basis to understand the key drivers of toxic emissions, and their temporal contributions to the toxicological footprint. This study adopts an input-output structural decomposition analysis (SDA)^10^,^11^ that combines U.S. economic, population and the National TRI datasets to understand what has driven toxic chemical release across a 16 year period between 1998 and 2013.

Our analysis quantifies the contribution of: population growth; changes in consumption volume induced exclusively by changes in per capita consumption of goods and services; consumption structure which is a function of shifts in consumption pattern or the types of goods and services being consumed; adjustments in production structure or the mix of inputs (for example, labour, domestic and imported materials) required to produce U.S. goods and services; and changes in emissions intensity or the total sectoral toxic release per inflation-adjusted unit of economic output.

To unravel and quantify the drivers of U.S. toxic chemical release, we introduce the concept of the toxicological footprint (TF) - a consumption-based^12^,^13^ indicator of toxic chemical release into the environment which accounts for all toxic emissions (based on the TRI) along entire domestic supply chains. This approach allows identification of the underlying drivers of the U.S.’s TF and the extent of their contributions to the overall changes in the toxic chemical release profile. A lack of understanding of these drivers hinders the opportunity for ascertaining the effectiveness of policies and the design of highly efficient supply chain networks induced by improvements in production efficiency through technological and socio-economic factors, in mitigating the effects of toxic emissions. The impact that toxic emissions have on climate change and human health combined with the absence of relational quantitative insight underpin the timeliness of this contribution.

## Sectoral toxic chemical release in the U.S. and derivation of toxic emissions intensity

All the raw toxic chemical release data set used in deriving the direct emissions intensity which forms the basis for the current work were obtained from the Toxics Release Inventory (TRI) of the U.S. The data captured within the TRI are for chemicals alone. Reporting is currently mandatory for individual manufacturing facilities in specified industries within the U.S. for over 600 chemicals and most cover environmental releases of each chemical, the medium of release (i.e. air, water, land and underground discharges of several thousand of toxic chemicals^14^,^15^) and facility characteristics. Other air pollutants or toxic releases including sulphur dioxide, carbon monoxide, nitrogen oxide and volatile organic compounds are not captured within the TRI. For detailed information on the data set and its associated limitations, see supplementary information (SI). The direct emissions intensity data covering 1998–2013 were derived from the TRI of the U.S.; see SI for details of how the toxic emissions intensity data were calculated. The data used are only for the chemicals in the TRI release. These datasets are reported in pounds (lb) of discharge, without any consideration given to the differences in toxicity among the chemicals, but the data provides the amount of discharge of the most important toxic chemicals.

## Toxicological footprint of key economic sectors in the U.S.

**Figure 1:**
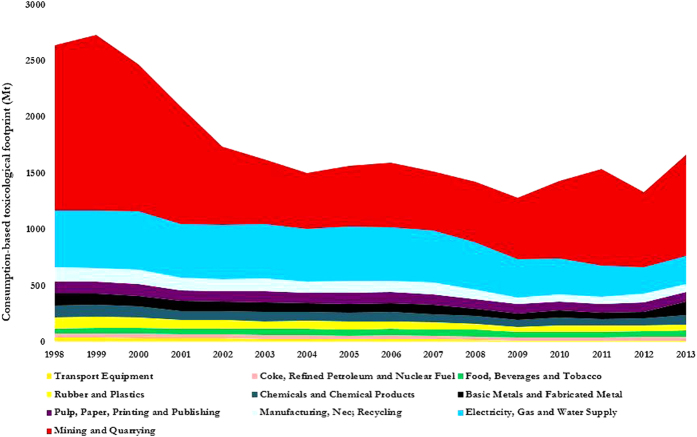
Contributions of the top 10 most intensive economic sectors to the total toxicological footprint (TF) of the U.S. between 1998 and 2013. As indicated, the mining and quarrying sector exhibit the most prominent sector responsible for the toxicological footprints of the U.S.

By linking the sectorial direct toxic emissions intensities matrix with total supply chain requirements across the 35 industries within the U.S. and final demand for goods and services, the toxicological footprint (TF) of the U.S. between 1998 and 2013 are calculated; refer to Methods Section and the SI for the methodological notes. Figure 1, shows TF for the top 10 economic industries. As indicated, the total TF decreased by approximately 37% (1000.08 megatons of toxicological footprint, Mt TF) from 2713.14 Mt in 1998 to 1713.06 Mt in 2013.

## Overall trajectory of the U.S. toxicological footprint from 1998 to 2013

**Figure 2:**
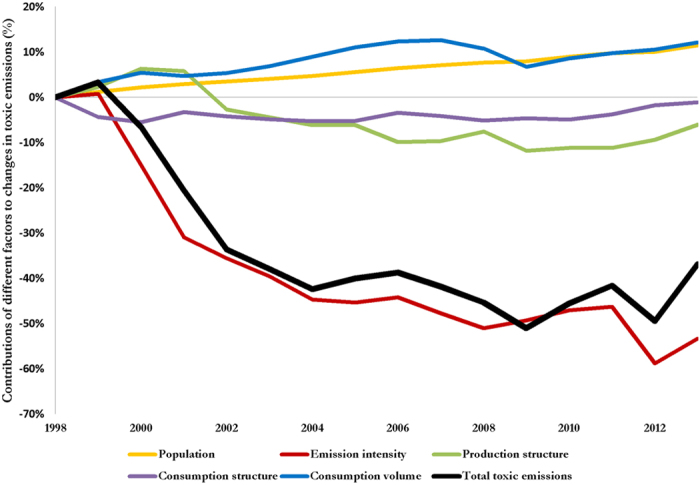
Contributions of different factors to changes in the U.S. toxicological footprint (TF) between 1998 and 2013. Using 1998 as a base year, the fat black line depicts the percentage change in total TF. The other trend lines show the contribution to the change in TF from population (yellow), consumption volume (blue), production structure (green), consumption structure (purple) and emissions intensity (red).

Figure 2 shows the overall trajectory of the U.S. toxicological footprint (TF) and the contributions of different sectors between 1998 (the baseline year) and 2013. After the surge in TF by 3.3% (88.4 Mt) between 1998 and 1999, driven by consumption volume (3.4%, 91 Mt), production structure (2.3%, 62 Mt) and population growth (1.2%, 32 Mt), the overall TF of the U.S. declined by 39% (1089 Mt) between 1999 and 2013. Three factors: (i) continued and steady growth in population (10.3%, 280 Mt); (ii) consumption volume triggered by per capita consumption (8.8%, 237 Mt) and (iii) consumption structure (3.2%, 88 Mt) acted as accelerators of the toxic release dynamics. However, the upward influence of these three factors was offset by a decline in emissions intensity (−54.1%, 1467 Mt) and production structure (−8.3%, 226 Mt).

**Figure 3:**
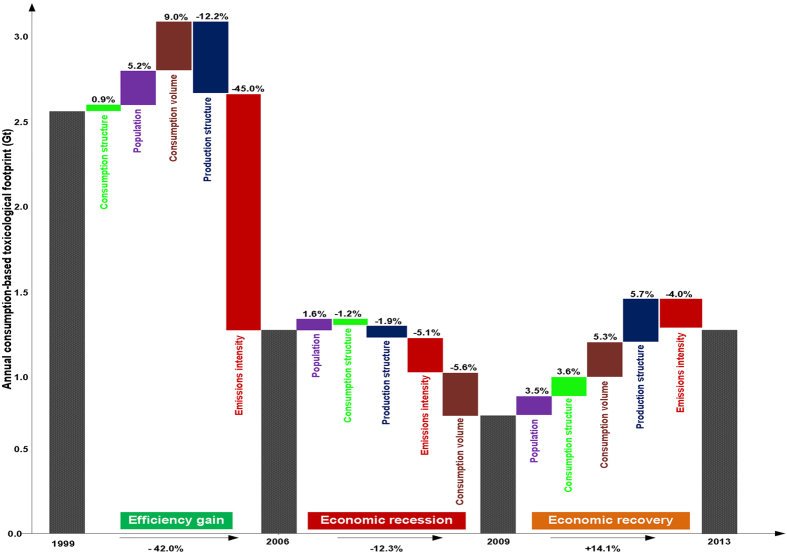
Contributions of different factors to the overall reduction in U.S. toxicological footprint (TF) based on three distinct categorical periods 1999–2006; 2006–2009 and 2009–2013. Each of the grey bars in each period represent the TF in gigaton. As shown, TF fell by 42.0% from 1999 to 2006 driven by efficiency gain, decreased by a further 12.3% between 2006 and 2009 due to global economic recession and increased by 14.1% between 2009 and 2013, driven mainly by economic recovery.

Despite an increase in population growth and consumption volume, and shift in consumption pattern, our results show improvements in emissions intensity drive the fluctuations pattern of TF. This trend demonstrates where technology (i.e. improved efficiency in production) directly mitigate toxic chemical release and by extension climate change due to reduced TF. To establish the overall strength or influence of toxic emissions intensity on TF across the time frame, we calculated the correlation coefficients (*r*) between these variables as 0.81. Thus, when the toxic emissions intensity (i.e. production efficiency) worsens in the U.S., TF increases. Efficiency gain was the main factor that drove down the TF during the 1999–2013 period, but other factors which dominated over shorter periods (See Fig. 3) are also discussed.

## Declining toxicological footprint from 1999 to 2009

Based on the aggregate time interval of declining TF between 1999 and 2009, population growth and increased consumption volume increased the TF by 10.2% (280 Mt) (6.8%, 190 Mt and 3.4%, 100 Mt respectively), offset by an improvement in efficiency (−50%, −1360 Mt) resulting in an actual decrease in TF of −54.2% (1470 Mt), Fig. 3. This latter effect was prominent between 1999 and 2006, where efficiency gains (i.e. improvement in emissions intensity) were responsible for 79% (1200 Mt) reduction in TF with changes to production structure responsible for 21% (330 Mt). Consumption structure, population growth and consumption volume exerted upward influence on TF during the same period. Overall, the TF decreased significantly by ~42% (1140 Mt) between 1999 and 2006. Almost half (41%, 150 Mt) of the toxic emission reduction between 2006 and 2009 was due to a sharp decrease in the volume of consumed goods as a result of decline in per capita consumption during the global economic recession (Fig. 3, brown bar). In particular, sharp decreases in the volume of capital expenditures and exported goods between 2007 and 2009 reduced emissions^10^.

**Figure 4:**
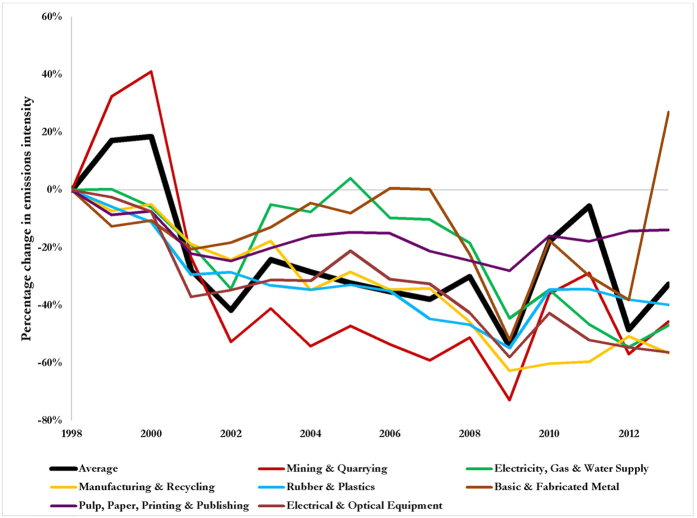
Percentage changes in the toxic emissions intensity of key sectors in the U.S. economy. See Supplementary Figure 2 for plots of trends in emissions intensity in absolute terms. As shown, the mining sector (red curve) is responsible for the largest improvements in toxic emissions intensity, relative to the weighted average of the toxic emissions intensity (black curve).

Given the influence of emissions intensity on the TF dynamics, we explored production and supply chain efficiencies to identify which industrial sectors are responsible for improvements or loss, and identify the factors upon which they depend. Figure 4 shows the percentage changes in the emissions intensity of key economic sectors where significant improvements in efficiency in terms of toxic chemical release per U.S.$ of output have been recorded, driving down the overall TF of the U.S. economy over the period 1998 to 2013. As shown, all sectors experience a relatively similar fluctuating pattern.

**Figure 5:**
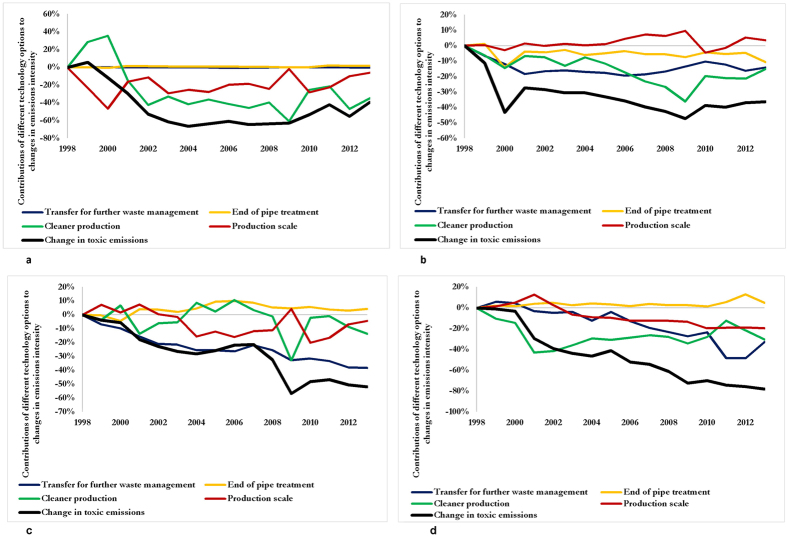
Contributions of four categories of mitigation options to reductions in toxic emissions intensity in the U.S. across key economic sectors, 1998–2013. Shown are changes in emissions intensity related to (**a**) mining and quarrying; (**b**) chemical products; (**c**) basic and fabricated metals; (**d**) electrical and optical products. In each panel, the solid black line indicates the percentage in emissions intensity driven by the mitigation options namely transfer for further waste management (blue), end of pipe treatment (yellow), cleaner production (green) and production scale (red).

Every economic sector has its specific characteristics. In our study, mitigation options for the specific management of toxic chemical release, driving the reduction in toxic emissions intensity were categorised into the following: (i) cleaner and efficient production processes (CP); (ii) end-of-pipe (EoP) treatment which involves the installation of filters like drains or smokestacks in the last process of production to prevent pollution; (iii) transfer for further treatment based on efficient waste management techniques, both on-site and off-site, including recycling, energy recovery and waste treatment (TFT); and (iv) production scale (PS) (i.e. sectoral output at deflated price). These four options were further structurally decomposed to quantify their contributions towards improvements in emissions intensity. We refer readers to Methods Section and SI for details of how the aforementioned options were quantified based on TRI datasets. Figure 5 shows the contributions of the mitigation options to reductions in toxic emissions intensity across key economic sectors.

The mining & quarrying (M&Q) sector is responsible for the largest release of toxic chemicals and heavy metals into the environment, Fig. 1. The same sector is responsible for the largest improvements in toxic emissions intensity (Fig. 4, red curve). For instance, the emissions intensity (i.e. gain in production efficiency) of the M&Q sector improved tremendously by 66% from 1999 to 2006. CP (green curve) and PS (red curve) are the dominant climate change mitigation options responsible for the improvements in emissions intensity in the M&Q sector, Fig. 5a. Improvements in emissions based on CP was due to technology and methods for efficient and cheaper extraction. Examples include innovation in refs 16 and 17: (i) exploration (i.e. identification of minerals, chemical compositions and physical properties directly in the field); (ii) ore deposit definition (i.e. modelling mineral deposits, their potential economic assets and challenges from the earliest stages of exploration); (iii) ore extraction; (iv) transport and communication; (v) ore processing; (vi) health and safety and (vii) remediation.

**Figure 6:**
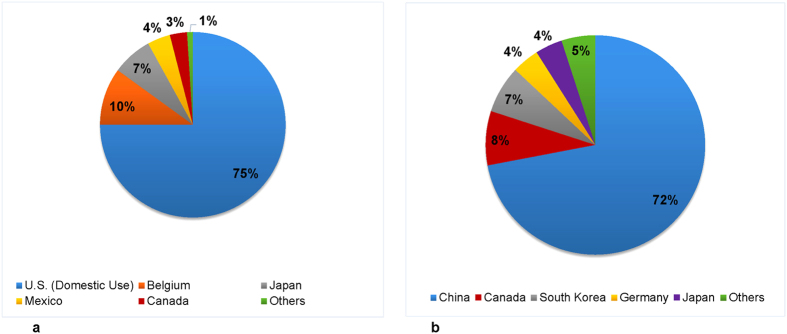
The destination of mined lead ore for processing. (**a**) destination of mined lead ore for processing in year 2000; (**b**) destination of mined lead ore for processing in 2014^21^,^54^. As indicated, in year 2000, 75% of lead ore mined in the U.S. was processed domestically, but in recent years more than 70% was exported to China and the remainder to the RoW for processing.

To explain the influence of production scale on improvements in emissions intensity in the M&Q sector, we explored trade data including export and import data of relevant items in the U.S. and found that it was largely due to outsourcing of the processing of hazardous chemicals to other parts of the world^18^,^19^. The U.S. in recent decades, has gradually shifted the most polluting aspects of its M&Q sector to developing countries, particularly lead ore. Almost all of the lead ore mined in the U.S. are exported for processing in countries whose environmental and occupational regulations are weaker or poorly enforced^20^,^21^. For example, 75% of the lead ore mined in the U.S. was processed and used domestically in 2000 with 25% exported to the rest of the world (RoW), whereas in 2014, 72% and the remaining 28% of the lead ore were outsourced to China and the RoW respectively (Fig. 6, see also Supplementary Figure 3).

For mining, outsourcing has become integral allowing mining companies to reduce production and transaction costs. Considerable capital is invested in constructing smelters and refineries and, along with energy costs, have encouraged outsourcing of processing-related mining activities. Based on U.S. Environmental Protection Agency’s new Clean Air rules^22^,^23^, it would require ~$100 m in new equipment, hence lead smelting lead ore process has been outsourced. In addition to environmental regulation governing mining in the U.S., lack of talent, an aging workforce, unfavourable tax laws, market structure, wage and price differentials have prompted outsourcing of mining processes^24^. Although the U.S. Federal income tax structure supports the development of the mining sector, the U.S. still has an unfavourable business tax environment in comparison to those countries likely to obtain outsourced mining related activities. For example, effective tax rate of the U.S. is about one-third higher than the average Organisation of Economic Cooperation and Development (OECD) countries. Non-wage costs including health insurance, payroll taxes, cost of litigation and exchange rate are some other factors. For instance, some processing of mined ore outsourced to Canada are due to the favourable exchange rate. In particular, established supply system, large engineering capacity and attractive labour force, encouraging environmental policies, low effective tax rates, competitive costs of raw materials and strong supplier networks in China are some of the motivating factors that prompted the U.S. to outsource large mining activities, Fig. 6b.

Improvements in emissions intensity in the M&Q sector is the most important driver in TF reduction, caused by a combination of outsourcing and more importantly cleaner production however, improvement in technology in other economic sectors also contributes. For instance, in the chemical sector, between 1999 and 2006, CP (−11%, 2,816 Mt), EoP (−5%, 1,280 Mt) and TFT (−13%, 3,327 Mt), Fig. 5b, contributed to reduction in toxic emissions of 24% (25,596 Mt). Similarly, between 1999 and 2006, in the basic and fabricated metal sector (Fig. 5c), TFT (blue curve) was the main climate change mitigation option responsible for an 18% reduction (2618 Mt). In the basic and fabricated metal sector, recycling rates of metals have increased and coupled with new and advanced technologies have reduced the need to extract virgin materials. This can be evidenced from the expansion of the low carbon and environmental business in the 1990 s which increased the value added of waste management and remediation services in the U.S. from $29.3 billion to $37.8 billion^25^,^26^. This investment stimulates companies to transfer their industrial waste to off-site plants rather than treatment on-site^27^. Of the 51% (3576 Mt) reduction in toxic emissions in the electrical and optical equipment sector (Fig. 5d), between 1999 and 2006, CP (green curve) and TFT (blue curve) have the greatest influence, each contributing 18% (643.7 Mt) and 19% (679.5 Mt). CP contributed to overall improvements in toxic emissions through improvement in production processes and product design to save on inputs derived from chemical substances in all sectors, Fig. 5. For instance, MidAmerican Energy succeeded in re-engineering their rubber and plastics manufacturing process through the use of advanced additives and ingredients, reducing toxic emissions intensity. Also, Xerox invested in chemical material substitution processes to minimise heavy metals and toxic chemicals. They migrated to a solvent-free process for the cleansing of machine parts across 15 years and succeeded in eliminating mercury and lead from their new range of products^27^.

## Growing toxicological footprint from 2009 to 2013

Between 2009 and 2013, as the economy recovered from recession, the U.S. TF surged by 14.1% (383 Mt) (Fig. 2, black curve). Economic recovery is reflected by the combined effects of production structure (5.7%, 155 Mt), consumption volume (5.3%, 145 Mt), consumption structure (3.6%, 97 Mt) and population growth (3.5%, 95 Mt) which nullifies the influence of 4% (110 Mt) improvement in emissions intensity. The growth in population, consumption volume caused by growth in per capita consumption and corresponding improved production activities in toxic emission intensive sectors of the economy resulted in an increase in the TF of the U.S. (see Supplementary Table 1). In this period, domestic raw and recycled materials were used to process aluminium, copper, brick, fertilizers, and steel and net imports of processed materials (worth about $28 billion) were consumed by downstream industries^28^. Also, within this time frame, the share of U.S. consumption of manufactured goods increased compared to services^10^, suggesting that changes in the types of goods and services consumed over time influence emissions^29^ and that it is not as straightforward as the balance of manufactured goods and services. These results show climate change impact from increased TF as a result of the growing economy.

## Discussion and Conclusion

Between 1999 and 2006, the TF of the U.S. decreased by 42% mainly driven by improvement in emissions intensity (i.e. gain in production efficiency) in the M&Q sector. Our analysis shows that in the same period, despite growth in population and increased consumption volume, improvements in emissions intensity through CP, EoP, and TFT prompted by effective environmental regulations reduced the TF in the U.S. Environmental outsourcing as a mitigation strategy leaking toxic chemical releases should also be addressed, alongside radical innovations in CP, EoP, TFT and PS. This efficiency gains which drove down TF has demonstrated quantitatively that reduction of TF is possible despite growth in population and consumption volume. For instance, CP contributed to the reduction of all types of toxic chemical release in all the sectors, consistent with the findings of Bui and Kapon^30^ and Fujii and Managi^27^. It can be concluded therefore, that legislation such as the 1990 Clean Air Act in the U.S. has been a positive technology-based or performance-based approach to enable large and small industrial sources of toxic chemical releases to adopt cleaner processes.

The relatively large decrease (−12.3%) in TF of the U.S. between 2006 and 2009 was mainly due to economic recession. The recession may also have contributed to efficiency gains in production structure. Since 2009, recovery in the U.S. increased the TF, but improvement in emissions intensity was overwhelmed by the combined effects of population growth, consumption and production structures as well as consumption volume. In fact, between 2009 and 2013, the TF of the U.S. increased by an average of 2.9% per year. This suggests that demand-side mitigation such as change in behaviour and consumption pattern, is needed to ensure a steady decline in the overall TF.

In addition to understanding the drivers of the U.S.’s TF dynamics during 1998–2013, our analysis assesses the efficacy of different drivers to reduce TF in the future. Efficiency gain, economic recession and economic recovery patterns which are coloured by population growth, change in consumption volume, production structure and emission intensity have provided the strong narratives in explaining why and how TF trajectory change in these reported 16 years for the first time. Although our results show the prominence of toxicological footprint emanating from the M&Q sector, should a more sectorial focus approach or even an international, a national, an organizational or a product level approach, be designed to address TF reduction? This has wider policy implication for multi-region debates on environmental toxicity.

This study represents the first structural decomposition analysis that unravels the underlying drivers of toxicity and the extent of their impact on climate change. Such analysis is helpful for policy and decision makers to understand the structural dynamics of toxic chemical release to formulate effective toxic chemical release mitigation standards and management protocols. Based on this research and to sustainably mitigate impact on climate change from long-lasting and pervasive toxic chemical release we recommend: (i) further research on more advanced treatment plans, systems of production that do not use polluting agents and remediation technology; (ii) provision of incentives for research and development to ensure the implementation of more efficient techniques to draw down TF and (iii) publishing detailed standards and guidelines tailored towards individual chemicals given that different toxic chemicals have different effects.

## Methods

### Input-output analysis

The general input-output (IO) model is a quantitative technique that is employed to examine the interrelationships between economic sectors over a time frame. It interprete the flows of goods and services from one economic sector (i.e. producer) to other economic sector (consumers)^31^. The basis of an IO analysis is the IO table which is made available by national government and takes the form of a square matrix which illustrates the financial input of products in dollars (as in the case for U.S.) from each sector of the economy (row) required to produce total output of each industry sector (column) also expressed in dollars^32^. Final demand (y) are demands for products in $ used by household, government, export etc. Total output is the dollar equivalent of outputs produced by each industry. Accordingly, the interdependencies between suppliers and consumers along the production chain across upstream and downstream industries within an economy and between economies^33^.

The relationship between the three variables within the framework of economic IO analysis is given by:


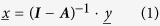


Matrix ***I*** is the identity matrix and (***I*** − ***A***)^−1^ known as the Leontief inverse matrix, ***L*** and represents the production structure. It shows that if ***L*** remains constant over a given period of time, the changes in total output (X) depend on the changes in final demand (Y). The implication of this equation is that sector *i* is required to generate an equivalent amounts of product to meet changes in final demand *Y.* As such, the outputs from other sectors to fulfil the additional requirements from sector *i* are also taken into account. This implies that it produces a mapping between the final demand vector and the inputs.

By adding environmental information, such as toxic emissions as in the case of the current work, to each sector, an environmental burden (a “footprint”) can then be assigned to these financial transactions. This characterises the environmental impact of an additional $1 of output from each industry. Environmental input-output analysis (EIO) illustrates economy-wide environmental repercussions (here we use toxic emissions as environmental indicator) triggered by economic activity, and can be expressed mathematically as:


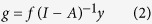


where g is the total economy-wide toxic release; f is a row vector of toxic coefficients (toxic release per unit of economic output) in each economic sector; I is the identity matrix; A is a matrix, and each column of A shows input requirement from each sector to produce one unit output of this column sector; y is a column vector of final consumption. For further information on how EIO framework is expanded upon and adopted the current work, see SI.

### Structural Decomposition Analysis (SDA)

Structural Decomposition Analysis (SDA) is a method frequently used to calculate the contribution of different factors to the overall change in carbon emissions and energy consumption. The SDA overcomes many of the static features of input-output models, enabling the evaluation of changes over time in economic structure, final demand components and categories. SDA is capable of distinguishing production and final demand effects that the Index Decomposition Analysis approach lacks^34^, and assesses direct and indirect effects along the entire supply chain across upstream and downstream industries^33^. Although the high level of data requirement by the SDA approach has been a barrier in the past since many countries publish input-output tables only once every 5 or more years, the recent development of global time series input-output databases (e.g. World Input-Output Database or WIOD^35^ and The EORA multi-region IO database^36^) and more regular publication of economic-structure data in countries like the U.S. now make time series SDA feasible and has been used in Australia^37^, Denmark^38^,^39^, India^40^, Korea^41^, Netherlands^42^, the United States^43^ and China^34^,^44^,^45^,^46^.

We consider the production structure through L = (I – A)^−1^, the Leontief inversion matrix. Changes in the production structure thus refer to changing input requirements of each sector or, in other words, industries using more or less intermediate inputs from each other. To distinguish the contributions of different final demand components, we further decompose y into three components – average consumption structure, per capita consumption volume, and population: **y = y**_**s**_*y*_*v*_ *p*, where **y**_**s**_ is a vector of per capita consumption patterns; *y*_*v*_ is a scalar of per capita consumption volume; *p* is a scalar of population which could appear at the front or the back of the input-output equation. Therefore, Eq. 2 can be transformed to:


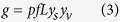


Over a given period of time, any changes in toxic footprint (Δ*TF*) in a country can be represented by Eq. 3, in which the five factors of population, toxic release intensity, production structure, consumption patterns, and consumption volume fully account for the changes in toxic release. A total difference of Eq. 3 generates Eq. 4


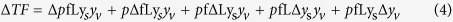


where, **∆** is the difference operator. Eq. 3 converts five multiplicative terms in the first term of Eq. 3 into five additive terms. Each additive term in Eq. 4 represents the contribution to a change in toxic release triggered by a factor assuming all other factors are constant. For example in the fifth term, **Δy**_**V**_ is change in per capita consumption volume, and the term represents the change of total toxic release caused by a change in per capita consumption volume, with population size, toxic release intensity, production structure, and consumption patterns staying constant.

In SDA, it is possible to compare different terms relative to any time point within a study period. However, there is no unique solution for the decomposition. We use the average of all possible first-order decompositions suggested by Dietzenbacher and Los^47^ and Seibel^48^. The U.S. input-output tables (IOT) from 1998 to 2013 were collected from the Bureau of Economic Analysis (BEA) which is in make-use format^49^. We convert the make-use table to symmetric IO table following the method by Miller and Blair^33^ and then aggregated them into 35 economic sectors to match the toxic release data. For further details on structural decomposition analysis, see SI.

### Logarithmic Mean Divisia Index (LMDI)

Given that improvement in emissions intensity induced by technology options was the main driver in reducing toxic emissions, it is important to evaluate how various technology options contributed towards the reduction. Accordingly, following the approach adopted by Fujii and Managi^27^ we further explored the TRI datasets based on three variables for toxic chemical release namely total chemical releases (**E**_**t**_); total off-site transfers for further waste management (**T**_**off**_); and on-site waste management (**T**_**on**_). We further established two additional variables namely: total amount of chemical substances generated (G), calculated as **G** = **E**_**t**_ + **T**_**off**_ + **T**_**on**_ and waste transferred out of the facility(**O**), computed as **O** = **E**_**t**_ + **T**_**off**_. **X** is the sectoral output data at constant dollar.

Based on the above generated and calculated dataset, Logarithmic Mean Divisia Index (LMDI) technique was adopted to decompose the changes in toxic emissions intensity using four factors namely (i) cleaner production (CP); (ii) end-of-pipe treatment (EoP); (iii) transfer for further waste management (TFT); and (iv) production changes (PC). We defined the CP as 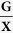, which is a function of toxic chemical release per gross output. This indicator can be decreased through the reduction of toxic chemical release whilst maintaining the same production output. This reduction can be achieved through improvement in production process and product design which can lead to overall reduction in the input of intermediate chemical materials. The EOP indicator was computed using the relation 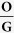, which is a function of the proportion of the out-of-facility chemical releases with respect to the total toxic chemical release during production. The reduction of this indicator can be realised by increasing the share of on-site waste management within the total toxic chemical releases.

The TFT indicator was calculated using the formula 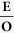, which is a function of the proportion of emissions with respect to the amount of toxic chemical release outside of the manufacturing facility (i.e. the summation of the toxic emissions and the amount transferred for further treatment). This indicator can be reduced if the total amount of toxic emissions released decreases and off-site waste management increases. Finally, the PC indicator is a function of change in production. Given the difficulty in obtaining the correct dataset based on individual product type, we employed the total sectoral output data to evaluate this variable.

The total amount of chemical substances emission (E) was decomposed as Eq. (5).


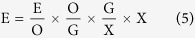


To decompose the emission change factor, the LMDI which has also been applied in several energy studies^50^,^51^,^52^,^53^ is adopted in this study as expanded upon in Eq. 6


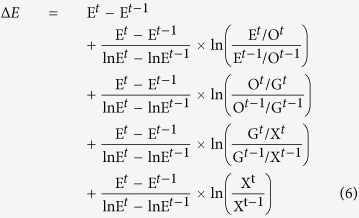


Accordingly, changes in the total toxic chemical emission (Δ*E*) were composed of the changes in TFT (first term), EOP (second term), CP (third term) and PC (fourth term).

## Additional Information

**How to cite this article:** Koh, S. C. L. *et al*. Drivers of U.S. toxicological footprints trajectory 1998–2013. *Sci. Rep.* **6**, 39514; doi: 10.1038/srep39514 (2016).

**Publisher's note:** Springer Nature remains neutral with regard to jurisdictional claims in published maps and institutional affiliations.

## Supplementary Material

Supplementary Information
