# Supplementary material for: Drivers of U.S. toxicological footprints trajectory 1998–2013
Source: Sci Rep. 2016 Dec 21;6:39514. doi: 10.1038/srep39514 (PMC5175177; doi:10.1038/srep39514)
Supplement: Supplementary Information [file srep39514-s1.pdf]

Drivers of US toxicological footprints trajectory 1998 - 2013

Koh, S.C. L[1, 2*], Ibn-Mohammed, T.[1, 2], Acquaye, A.[3], Feng, K.[4], Reaney, I.M.[5], Hubacek, K.[4], Fujii, H.[6], Khatab, K.[7]

[1] Centre for Energy, Environment and Sustainability, University of Sheffield, Sheffield, S10 1FL, UK.
[2]Advanced Resource Efficiency Centre, University of Sheffield, Sheffield, S10 1FL, UK.
[3]Kent Business School, University of Kent, Canterbury, CT2 7PE, UK.
[4]Department of Geographical Sciences, University of Maryland, College Park, Maryland 20742, USA. [5]Departments of Materials Science and Engineering, University of Sheffield, Sheffield, S1 3JD, UK. [6]Graduate School of Fisheries and Environmental Sciences, Nagasaki University 1-14 Bunkyo-machi, Japan.
[7]Centre for Health and Social Care Research, Sheffield Hallam University, Sheffield, S10 2BP, UK.

* Corresponding author (S.C.L.Koh@sheffield.ac.uk/t.ibn-mohammed@sheffield.ac.uk)

Supplementary Information

This document includes:

- Supplementary Data and Methods
- Supplementary Analysis
- Supplementary References

1.0 Supplementary Data and Methods

1.1 Derivation of environmental emissions intensities of toxicology for Input-Output analysis

The environmental extension (i.e. the direct emissions intensity) matrix (DIMS) for toxic releases in the US does not exist. As such in this paper, a newly developed set of data was originally derived from the Toxic Release Inventory for the US [1]. The direct emissions intensity data covering 1998 -2013 were derived from the Toxic Release Inventory (TRI) of the U.S which provides toxic release estimates, including air, water, land and underground, for a number of toxic chemicals[2]; and the US national economic input-output table which provides information on economic activities. These data sets are reported in pounds (lb) of discharges, without any consideration given to the differences in toxicity among the chemicals, but the data provides indications of discharges of the most important toxic substances that is attributed to a particular manufacturing facility[1].

The classifications of economic sectors in the US are based on North American Industry Classification System (NAICS)[3] and is consistent with the Standard Industry Classification (SIC) codes used on the TRI database for the recordings of toxic discharge. However, on the TRI database, the SIC are not categorised into 35 economic sectors but rather as a general pool of several categories of SICs. Hence it was important to map the SIC with the appropriate economic sectors, ensuring consistency with the NAICS format. Accordingly, the economic sectors (based on SIC) and toxic discharges (reported in pounds for all chemicals captured in the TRI) are mapped in the following format: Agriculture, Hunting, Forestry and Fishing (SIC 111110 - 115310); Mining and Quarrying (SIC 211111 - 213115); Food, Beverages and Tobacco (SIC 311111 - 312230); Chemicals and Chemical Products (SIC 325110 -325199); Basic Metals and Fabricated Metal (SIC 331110 - 332999); and so on. This was intelligently carried out using advanced functionalities in Microsoft Excel Software. So, for consistency, the NAICS, which exist in a disaggregated form were mapped to conform to the 35 × 35 economic sectors of Bureau of Economic Analysis (BEA) which is in make-use format. Essentially, for each economic sector per year, the total toxic release (lb) is the sum of all the toxic discharges on air, water, land and underground. The direct intensity matrices (DIMs) were then calculated by dividing the total toxic release by the total sectoral outputs per economic sectors:

$$D_{IM} = \frac{\text{Total toxic release (kg)}}{\text{Total sectoral output (\$)}} \qquad (1)$$

In order to account for inflation on a year by year basis, we converted the sectoral output to constant dollar using the relation:

$$\text{Output at constant dollar} = \frac{\text{Total sectoral output}}{\text{Coefficient of pricing index}} \qquad (2)$$

The datasets for the coefficient of pricing index (CPI) was obtained from the Bureau of Economic Analysis (BEA) [4]. Accordingly, the direct intensity matrix after inflation is taken into account becomes:

$$D_{IM} = \frac{\text{Total toxic release (kg)}}{\text{Sectoral output at constant dollar (\$)}} \qquad (3)$$

To obtain the Total Intensity Matrix (TIMS) of the US across the economic sectors, we multiplied their respective Leontief inverse matrix by the US sectoral toxic intensities which was estimated as total kilogram of toxic release per dollars' worth of output, between 1998 and 2013. This newly developed data on sectoral toxic intensities (i.e. a measure of the efficiencies of the economic sectors in terms of toxicity) was then used within the structural decomposition analysis framework to investigate trends in the distribution of industrial toxic discharge (Supplementary Figure 1). The mathematical basis for this approach are detailed in the section that follows.

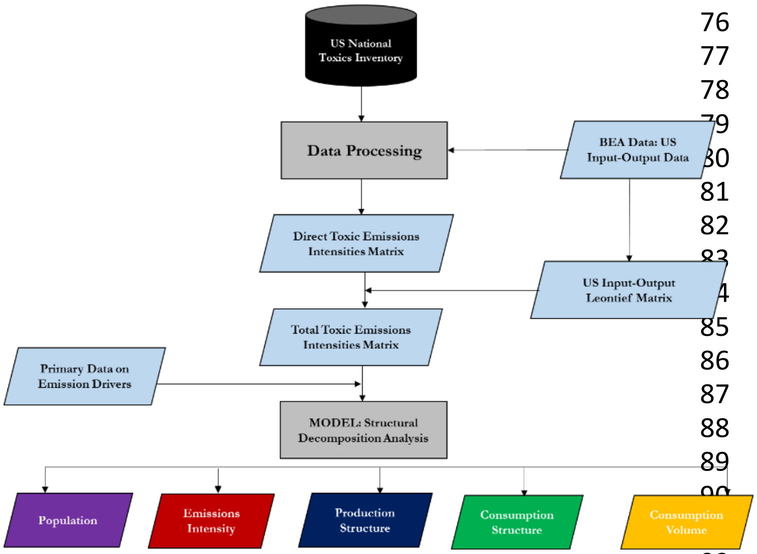

Supplementary Figure 1: Methodological framework for input-output structural decomposition analysis.

1.2 Data source and limitations

All the raw toxic chemical release data set used in deriving the direct emissions intensity which forms the basis for the current work was obtained from the Toxics Release Inventory (TRI) of the U.S. Individual manufacturing facilities in specified industries within the U.S. are mandated to report their air, water, land and underground discharges of several thousand of toxic chemicals[2, 5]. All data are reported in pounds of toxic chemical release. For example, a candidate chemical such as nitric acid is reported for both onsite and offsite releases, so that the total toxic release of nitric acid is the sum of both onsite and offsite releases, measured in pounds, into air, water, land and underground. With these sets of data, toxic discharge estimates are made with respect to a particular output changes by destination media as well as by specific toxic chemicals.

However, there are some limitations with the TRI datasets. For instance, not all manufacturing facilities, especially industries with small plants, are mandated to report their toxic chemical releases[5]. In the TRI, data are recorded in pounds of toxic discharges without any form consideration given to the dissimilarities among the chemicals. Also, most of the datasets within the TRI are mainly estimates as against actual measurements of the toxic discharges. As such, the quality of the estimates differs, with some manufacturing facilities endowed with the ability to report accurate values within a few percent on some chemicals, and some other manufacturing facilities may be less accurate by a significant margin in their reporting of toxic discharge[5] .

Additionally, the data captured within the TRI are for chemicals alone. Reporting is currently mandatory for over 600 chemicals and most cover environmental releases of each chemical, the medium of release (i.e. air, water, land, underground) and facility characteristics[3]. Other air pollutants or toxic releases including sulfur dioxide, carbon monoxide, nitrogen oxide and volatile organic compounds are not captured within the TRI. Similarly, within the TRI, there are no all-inclusive datasets on water pollution especially as it pertains to pesticides, biochemical oxygen demand etc[5]. Despite these limitations, the TRI data offer an indications of toxic releases of the most important toxic chemicals or materials that is enough for the type of analysis presented in the current work.

1.3 Economic input-output model

This section describes the methodological approach for computing the toxicological footprint of the US across the 35 sectors under consideration. An economic input-output (EI-O) model which requires several distinct calculations steps was used to estimate both direct and indirect changes in output throughout the economy for each of the 35 economic sectors. The I-O process utilises economic data of cash flow among various sectors of industry [6]. The data are organised into an I-O table made available by the national government. In the current work, the U.S. input-output tables (IOT) from 1998 to 2013 were collected from the Bureau of Economic Analysis (BEA).

The I-O table takes the form of a square matrix which illustrates the financial input in £ (as in the case for UK) from each sector **(row)** required to make £1 worth of output **(column).** Assuming an input-output table is organised into n sectors, the main input-output table is a $n \ x \ n$ matrix. Each cell of the matrix describes the deliveries between two particular sectors. The **rows** and **columns** of the matrix describe the supplying and receiving sectors respectively. The I-O table therefore contains three key aggregated information namely intermediate consumption (**Z**), Final demand (**Y**) in £ and Total output (**X**) in £. The intermediate consumption is the resources (input) required by a given sector of an industry to produce an output[7]. Final demand is demand used by household, government, export etc. Total output is the sum of all inputs (i.e. requirements) from each of the economic sectors needed to produce a unit output. Finally, environmental discharges (i.e. toxic release in lb., in this case) associated with both direct and indirect changes are then assessed by multiplying the output changes by the average level of toxic discharges.

1.3.1 Mathematical formulations

The relationship between Intermediate consumption (Z), Final demand (Y) and Total output (X) is given by:

$$\mathrm{Z} + \mathrm{Y} = \mathrm{X} \tag{4}$$

This can be written as:

$$\sum \mathrm{Z_i} + \sum \mathrm{Y_i} = \sum \mathrm{X_i} \tag{5}$$

Since industries purchase from other industries to produce their own goods and services, the I-O table is therefore used to determine these indirect deliveries (from one industry to another) by deriving a **technology matrix**, also known as a **matrix of direct requirement**. This is a matrix indicating sector-to-sector flows of purchases[7]. It is the requirement from each of the economic sector needed to produce a unit output and is denoted by A. Therefore, an entry $\mathrm{A_{ij}}$ represents the purchases from economic sector i associated with a £1 output from sector j.

Technology Matrix (A) is given by:

$$\mathrm{A} = \frac{\text{Intermediate consumption}}{\text{Total output}} = \frac{\mathrm{Z}}{\mathrm{X}} \tag{6}$$

Therefore

$$\mathrm{Z} = \mathrm{AX} \tag{7}$$

Substituting equation 7 into 4, we have

$$\mathrm{AX} + \mathrm{Y} = \mathrm{X} \tag{8}$$

So that the input-output system can be written as

$$\mathrm{Y} = \mathrm{X} - \mathrm{AX} = \mathrm{X}(1 - \mathrm{A}) \tag{9}$$

But A is a matrix, therefore Equation 9 can be re-written as:

$$\mathrm{Y} = \mathrm{X}(\mathrm{I} - \mathrm{A}) \tag{10}$$

Where, I is an identity matrix. Equation (10) can be re-written as

$$\mathrm{X} = (\mathrm{I} - \mathrm{A})^{-1}\mathrm{Y} \tag{11}$$

Equation 11 is the **Leontief Inverse Matrix**, named after Wassily Leontief, who developed the input-output analysis framework, for which he received a Nobel Prize in 1973. The interpretation of equation

11 is that by multiplying the sector of the change in final consumption by $(\mathrm{I}-\mathrm{A})^{-1}$, we get the total production required to attain the final output. The **Leontief Inverse Matrix** is the matrix of cumulative (direct and indirect) deliveries needed to produce a product per unit of total output and it can be approximated by the power series approximation of the matrix of direct requirement coefficient[7].

Expanding equation 11 to the infinite series of inter-sector transactions, we have

$$\mathrm{X} = (\mathrm{I} + \mathrm{A} + \mathrm{A}^2 + \mathrm{A}^3 + \mathrm{A}^4 + \cdots)\mathrm{Y} \qquad (12)$$

The total output (X) associated with the final demand (Y), can be defined as the first two terms in equation 9. By suppressing all indirect effects $(A^2, A^3, A^4, etc.)$, equation 9 becomes

$$\mathrm{X} = (\mathrm{I} + \mathrm{A})\mathrm{Y} \qquad (13)$$

Equation 13 represents the direct requirement from producers in order to allow the industry to produce the final output.

By adding environmental information, in this case, toxic chemical releases, to each sector, an environmental burden (a "footprint") can then be assigned to these financial transactions. This environmental impact characterises the toxic discharges into air, water, underground and land of an additional $1 of output from each industry.

Let $\mathbf{E} = \{\mathbf{e_{kj}}\}$ be the vector of environmental effect or environmental extension matrix (i.e. the toxic emissions or toxic discharge to produce the total output of each industry); **X** be the total output.

Then, $\mathbf{D_{IM}}$ Direct Intensity Matrix (i.e. the sectoral direct emissions intensities derived for toxic emissions k across j industries) is given by:

$$\mathrm{D_{IM}} = \frac{\text{Environmental Extension Matrix}}{\text{Total output}} = \frac{\mathrm{E}}{\mathrm{X}} \qquad (14)$$

$$\therefore \text{Total sector emissions } \mathrm{E} = \mathrm{D_{IM}}\,\mathrm{X} \qquad (15)$$

Substituting equation 11 into equation 15 yields:

$$\mathrm{E} = \mathrm{D_{IM}}.(\mathrm{I}-\mathrm{A})^{-1}\mathrm{Y} \qquad (16)$$

Let Total Intensity Matrix $\mathrm{T_{IM}} = \mathrm{D_{IM}}.(\mathrm{I}-\mathrm{A})^{-1} = \mathrm{D_{IM}}.(\mathrm{I}+\mathrm{A})$

$$\therefore \mathrm{E} = \mathrm{T_{IM}}.\,\mathrm{Y} \qquad (17)$$

Hence toxicological footprint (TF) in kg of toxic chemical release is given by the matrix multiplication of

$$\text{Total Intensity Matrix (kg/\$)} \times \text{Final demand (\$)} \qquad (15)$$

Thus, in an EIO analysis, the toxicological footprint is calculated by multiplying the final demand ($) of an economic sector by the toxic emissions intensity of that sector expressed in kg/$. In matrix notations, the final demand matrix would be a column matrix with dimension $(n\ x\ 1)$.

2.0 Supplementary methods on structural decomposition analysis

As presented in Materials and Methods, in this study the change of toxic chemical release is decomposed into five additive terms, and each term represents the contribution of the changing factor to the total change of toxic release in the US. One can perceive a logical pattern that the changing factors is placed, at each term, in turn from left to right in the product with all other factors; and the other unchanged factors on the left hand side of the changing factors are in base year value (year "*t – 1*"); and the ones on the right hand side of the changing factors are in target year value (year "*t*"). Therefore, by extracting the unchanged values in each term the equation can be merged as:

$$\Delta\mathbf{g} = \mathbf{w}^{p}\boldsymbol{\Delta}p + \mathbf{w}^{f}\Delta\mathbf{f} + \mathbf{w}^{L}\Delta\mathbf{L} + \mathbf{w}^{y_s}\Delta\mathbf{y}_s + \mathbf{w}^{y_v}\Delta y_v \qquad \textbf{(S1)}$$

where the w^{p}, w^{f}, w^{L}, w^{y_s}, and w^{y_v} are the so-called "weight" or "coefficient" for each "Δfactor" respectively. The calculation of these "weights" or "coefficients" are usually done via econometric methods; alternatively, they can be generated via a more straight forward way by deriving them with the structural decomposition method [8, 9].

However, equation (S1) is not a unique decomposition equation, which is only one of the 120 decomposition equations by assuming the order of the driving forces of "$\mathbf{p}\cdot\mathbf{f}\cdot\mathbf{L}\cdot\mathbf{y}_s\cdot y_v$". However, the order can also be "$\mathbf{f}\cdot\mathbf{p}\cdot\mathbf{L}\cdot\mathbf{y}_s\cdot y_v$" or "$\mathbf{f}\cdot\mathbf{p}\cdot\mathbf{L}\cdot\mathbf{y}_s\cdot y_v$" and so on. Although each decomposition equation would produce exactly the same result for Δg, de Haan [10] found that the size of the contribution of each "Δfactor" significantly differs across the equations. In other words, the "coefficient" (w) of each "Δfactor" is varied in different equations.

Due to the non-uniqueness issue, Dietzenbacher and Los [11] suggested to take the average of all the $n!$ ($5!$ in this case) decomposition equations (Supplementary Table S1). In order to do so, all the 120 equations need to be sorted into a standard order, for example, every term in the equation needs to be re-arranged to the order of "$\mathbf{p}\cdot\mathbf{f}\cdot\mathbf{L}\cdot\mathbf{y}_s\cdot y_v$", and the "Δfactor" is in turn placed from the first factor of "p" in the first term of the equation to the last factor of y_s in the last (seventh) term. Then, all the equations have been re-arranged in the same pattern. For example, the first term of every equation contains the information of the contribution of population growth (Δp) to the change of toxic release (Δg) with other factors kept unchanged. The product of the unchanged values of other factors is the "coefficient" for Δp. The "coefficient" $\mathbf{f}_{(t-1)}\cdot\mathbf{L}_{(t-1)}\cdot\mathbf{y}_{s(t-1)}\cdot y_{v(t-1)}$ appears 24 times, and same as the "coefficient" $\mathbf{f}_t\cdot\mathbf{E}_t\cdot\mathbf{L}_t\cdot\mathbf{y}_{st}\cdot y_{vt}$ does. de Haan [10] and Seibel [12] found that each term in the equation always has $2^{(n-1)}$ different "coefficients" attached to the "Δfactor", $2^{(5-1)} = 32$ different "coefficients" to every "Δfactor" in this case.

Next one can calculate the "weights" of the "coefficients" which is attached to the "Δfactor". The easiest way is via observations, to count how many cases of "Δfactor" are attached to the same "coefficient". For example as mentioned previously, the "coefficient" $\mathbf{f}_{(t-1)}\cdot\mathbf{L}_{(t-1)}\cdot\mathbf{y}_{s(t-1)}\cdot\mathbf{y}_{v(t-1)}$ appears 24 times in the 120 equations, and therefore its weight is 24. However, the observation method could be difficult in large number of decomposition equations with more than 5 factors.

Seibel [12] proposed a mathematic method to deal with this. Firstly, let k represent the number of subscript "$t-1$" (base year) in a coefficient; k runs from "0" to "$n-1$"; therefore, the number of subscript "t" (target year) would be "$n-1-k$". Secondly, for each k, the number of different coefficients attached to the "Δfactor" can be calculated by:

$$\frac{(n-1)!}{(n-1-k)!\cdot k!} \qquad \textbf{(S2)}$$

In this study, n is set to 5 (five factors). So when k=0 or 4, there is only one coefficient for each case; when k=1 or 3, the number of different coefficients are 4 respectively; when k=2, there would be 6 different coefficients. Thirdly, Equation (S3) calculates how many times each of these coefficients is repeated as "weights" for each "Δfactor" term in every equation of $n!$. The results for Equations (S2) and (S3) are shown in Table S1 for the case of $n = 5$

$$(n-1-k)!\cdot k! \qquad \textbf{(S3)}$$

Therefore, each "w" attached to the "Δfactor" in Equation S1 can be present, for example,

$$w^{p}\Delta p = \frac{1}{120}[(24\cdot\Delta p\cdot\mathbf{F}_{(t-1)}\cdot\mathbf{L}_{(t-1)}\cdot\mathbf{y}_{\mathbf{s}(t-1)}\cdot y_{v(t-1)}) +$$

$$(6\cdot\Delta p\cdot\mathbf{F}_{(t)}\cdot\mathbf{L}_{(t-1)}\cdot\mathbf{y}_{\mathbf{s}(t-1)}\cdot y_{v(t-1)}) +$$

$$(6\cdot\Delta p\cdot\mathbf{F}_{(t-1)}\cdot\mathbf{L}_{(t)}\cdot\mathbf{y}_{\mathbf{s}(t-1)}\cdot y_{v(t-1)}) +$$

$$(6 \cdot \Delta p \cdot \mathbf{F}_{(t-1)} \cdot \mathbf{L}_{(t-1)} \cdot \mathbf{y}_{\mathbf{s}(t)} \cdot y_{v(t-1)}) +$$

$$(6 \cdot \Delta p \cdot \mathbf{F}_{(t-1)} \cdot \mathbf{L}_{(t-1)} \cdot \mathbf{y}_{\mathbf{s}(t-1)} \cdot y_{v(t)}) +$$

$$(4 \cdot \Delta p \cdot \mathbf{F}_{(t)} \cdot \mathbf{L}_{(t)} \cdot \mathbf{y}_{\mathbf{s}(t-1)} \cdot y_{v(t-1)}) +$$

$$(4 \cdot \Delta p \cdot \mathbf{F}_{(t)} \cdot \mathbf{L}_{(t-1)} \cdot \mathbf{y}_{\mathbf{s}(t)} \cdot y_{v(t-1)}) +$$

$$(4 \cdot \Delta p \cdot \mathbf{F}_{(t)} \cdot \mathbf{L}_{(t-1)} \cdot \mathbf{y}_{\mathbf{s}(t-1)} \cdot y_{v(t)}) +$$

$$(4 \cdot \Delta p \cdot \mathbf{F}_{(t-1)} \cdot \mathbf{L}_{(t)} \cdot \mathbf{y}_{\mathbf{s}(t)} \cdot y_{v(t-1)}) +$$

$$(4 \cdot \Delta p \cdot \mathbf{F}_{(t-1)} \cdot \mathbf{L}_{(t)} \cdot \mathbf{y}_{\mathbf{s}(t-1)} \cdot y_{v(t)}) +$$

$$(4 \cdot \Delta p \cdot \mathbf{F}_{(t-1)} \cdot \mathbf{L}_{(t-1)} \cdot \mathbf{y}_{\mathbf{s}(t)} \cdot y_{v(t)}) +$$

$$(6 \cdot \Delta p \cdot \mathbf{F}_{(t-1)} \cdot \mathbf{L}_{(t)} \cdot \mathbf{y}_{\mathbf{s}(t)} \cdot y_{v(t)}) +$$

$$(6 \cdot \Delta p \cdot \mathbf{F}_{(t)} \cdot \mathbf{L}_{(t-1)} \cdot \mathbf{y}_{\mathbf{s}(t)} \cdot y_{v(t)}) +$$

$$(6 \cdot \Delta p \cdot \mathbf{F}_{(t)} \cdot \mathbf{L}_{(t)} \cdot \mathbf{y}_{\mathbf{s}(t-1)} \cdot y_{v(t)}) +$$

$$(6 \cdot \Delta p \cdot \mathbf{F}_{(t)} \cdot \mathbf{L}_{(t)} \cdot \mathbf{y}_{\mathbf{s}(t)} \cdot y_{v(t-1)}) +$$

$$(24 \cdot \Delta p \cdot \mathbf{F}_{(t)} \cdot \mathbf{L}_{(t)} \cdot \mathbf{y}_{\mathbf{s}(t)} \cdot y_{v(t)})]$$

And it is similar to obtain other "*w*"s in Equation (S1).

Table S1: Subscripts for the components of "Δfactor's" coefficients and their weights

k	Subscript for the components in the coefficients				**Weight**
	first	second	third	fourth	
**0**	*t*-1	*t*-1	*t*-1	*t*-1	**24**
**1**	*t*	*t*-1	*t*-1	*t*-1	**6**
	t-1	*t*	*t*-1	*t*-1	
	t-1	*t*-1	*t*	*t*-1	
	t-1	*t*-1	*t*-1	*t*	
**2**	*t*	*t*	*t*-1	*t*-1	**4**
	t	*t*-1	*t*	*t*-1	
	t	*t*-1	*t*-1	*t*	
	t-1	*t*	*t*	*t*-1	
	t-1	*t*	*t*-1	*t*	
	t-1	*t*-1	*t*	*t*	
**3**	*t*-1	*t*	*t*	*t*	**6**
	t	*t*-1	*t*	*t*	
	t	*t*	*t*-1	*t*	
	t	*t*	*t*	*t*-1	
**4**	*t*	*t*	*t*	*t*	**24**

3.0 Supplementary information on trend analysis

Supplementary Figure 2 shows the toxic emissions intensity profile of top 6 economic sectors in absolute values.

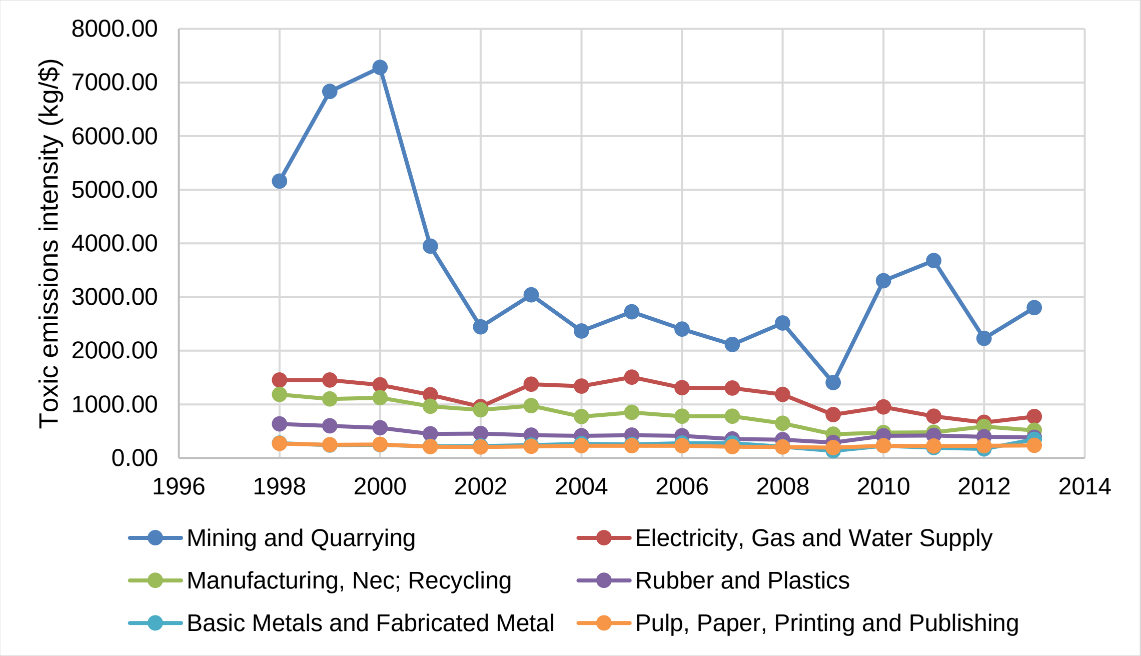

Supplementary Figure 2: Emissions intensity (toxic chemical release per dollar (US$) of output) of key sectors in the US economy (plots in terms of absolute value)

4.0 U.S. Lead Ore Exports (Megatonnes) to China

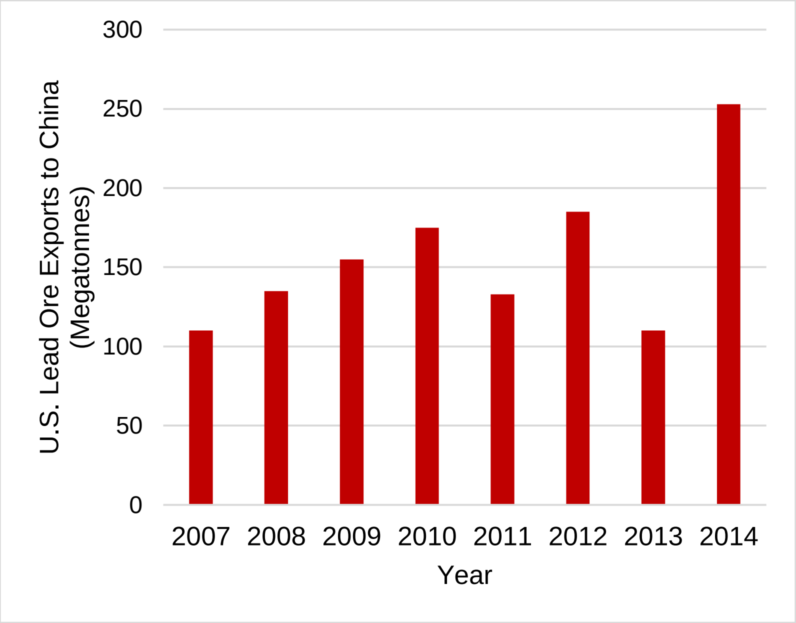

Supplementary Figure 3: U.S. Lead Ore Exports (Megatonnes) to China 2007-2014[13]

5.0 United States Manufacturing Output and Manufactured Goods Exports

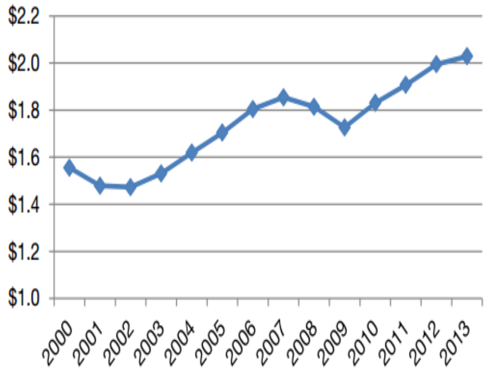

Supplementary Figure 4a: United States Manufacturing Output, in Billions of Dollars, (2000–2013) [4]

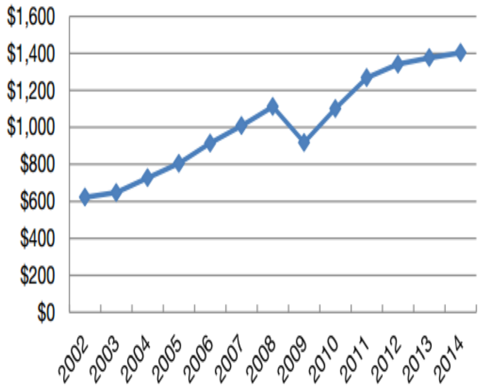

Supplementary Figure 4b: United States' Manufactured Goods Exports, in Billions of Dollars, (2000-2014) [4]

6.0 Production structure of the U.S. between 2007 and 2013

Supplementary Table 1: improved production activities in toxic emission intensive sectors in key economic sectors

Key economic sectors	2009	2010	2011	2012	2013
**Agriculture**	0.7%	0.7%	0.8%	0.8%	0.8%
**Mining**	0.3%	0.4%	0.5%	0.5%	0.5%
**Coke, Refined Petroleum and Nuclear Fuel**	0.8%	0.9%	1.2%	1.2%	1.3%
**Chemicals, rubber and other non-metal products**	1.6%	1.8%	2.0%	1.8%	1.9%
**Metal products**	1.0%	1.2%	1.3%	1.3%	1.3%
**Machinery and equipment**	2.3%	2.4%	2.6%	2.7%	2.8%
**Other manufacturing**	3.4%	3.4%	3.5%	3.5%	3.4%
**Electricity, Gas and Water Supply**	0.6%	0.6%	0.5%	0.4%	0.4%
**Construction**	1.8%	1.6%	1.5%	1.5%	1.5%
**Transportation**	2.7%	2.8%	2.9%	3.0%	3.0%
**Services**	22.2%	22.3%	22.0%	22.1%	22.1%
**Import**	4.1%	4.6%	5.1%	5.0%	4.7%

Supplementary references

1. TRI, TRI Basic Data Files: Calendar Years 1987 - 2013, http://www2.epa.gov/toxics-release-inventory-tri-program/tri-basic-data-files-calendar-years-1987-2013, Accessed 10th March 2015.
2. Ranson, M. *et al*. *Env. Science & Technology*, 49, 12951-12957 (2015).
3. NAICS, North American Industry Classification System, https://www.census.gov/cgi-bin/sssd/naics/naicsrch?chart=2012, Accessed 10th March 2015.
4. BEA, U.S. Bureau of Economic Analysis (2014).
5. Lave, L.B. *Env. Science & Technology*, 29, 420A-426A (1995).
6. Raa, T.T. *Economic Systems Research*, 19, 453-459 (2007).
7. Miller, R.E. & Blair, P.D. *Input-output analysis: foundations and extensions* (Cambridge University Press, 2009).
8. Hoekstra, R. & van der Bergh, J.C.J.M. *Env. & Resource Economics* 23, 357-378 (2002).
9. Guan, D. *et al*. *Geophysical Research Letters* 36, 1-5 (2009).
10. de Haan, M. *Economic Systems Research* 13, 181-196 (2001).
11. Dietzenbacher, E. & Los, B. *Economic Systems Research* 10, 307-323 (1998) .
12. Seibel, S. *Decomposition Analysis of Carbon Dioxide Emission Changes in Germany - Conceptual Framework and Empirical Results. European Commission* (2003).
13. U.S. International Trade Commission, Dataweb U.S. Import and Export, https://www.usitc.gov/ Accessed 10th February, 2016.
